# Intervention development and optimisation of a multi-component digital intervention for the monitoring and management of hypertensive pregnancy: the My Pregnancy Care Intervention

**DOI:** 10.1186/s40814-024-01562-9

**Published:** 2024-11-12

**Authors:** Katherine Tucker, Frances Rose, Layla Lavallee, Cristian Roman, Lucy Goddard, Richard J. McManus

**Affiliations:** 1 Nuffield Department of Primary Care Health Sciences, University of Oxford, Oxford, UK; 2 Institute of Biomedical Engineering, Department of Engineering Science, University of Oxford, Oxford, UK; 3 Brighton and Sussex Medical School, University of Sussex, Brighton, BN1 9PX UK

## Abstract

**Background:**

Hypertensive disorders of pregnancy affect around 10% of pregnancies and remain a major cause of maternal and foetal morbidity and mortality. Trials have shown that self-monitoring blood pressure during pregnancy is safe, but self-monitoring alone does not improve blood pressure control or pregnancy outcomes. This study aimed to develop and optimise a multicomponent intervention to support blood pressure monitoring, hypertension management and urine testing within current care pathways.

**Methods:**

Relevant literature, input from patient and public contributors (PPI) and stakeholder groups, and the researcher’s previous experience were used to develop an initial intervention. Think-aloud interviews and focus groups with women from diverse backgrounds with lived experience of hypertension in pregnancy and healthcare professionals provided feedback on the intervention prototype (*n* = 29). The MRC Framework for Developing Complex Interventions guided the processes to optimise the intervention’s acceptability and maximise engagement. A detailed tabulation of participants’ views and logic models was produced using the COM-B model of Behaviour Change.

**Results:**

The prototype intervention was acceptable and viable to both pregnant women with experience of hypertensive pregnancy and healthcare professionals. Emerging themes centred on how the intervention could be optimised within current National Health Service care pathways and the lives of pregnant women to support behaviour change. Key target behaviours to support the intervention included increasing understanding of blood pressure management, engagement with the intervention, monitoring blood pressure and urine and taking appropriate actions based on those readings. This informed the development of recommendations involving clear action timelines for women and evidence-based guidance to support decision-making by healthcare professionals. The findings were used to produce the multi-component *My Pregnancy Care* intervention, consisting of a smartphone application and an information leaflet to support blood pressure self-monitoring and proteinuria self-testing, self-management of antihypertensive medication and smartphone application use.

**Conclusions:**

This research provided comprehensive insight into the needs of pregnant women with hypertension and their healthcare teams regarding self-monitoring and management of blood pressure. This supported the development of a tailored multi-component digital intervention that addresses barriers to blood pressure self-management by being user-friendly, persuasive and acceptable. It is hoped that the intervention will support the monitoring and management process, collaboration between healthcare professionals and women, clinical action and improved clinical outcomes.

## Key messages regarding feasibility

- Previous trials have demonstrated that blood pressure self-monitoring during pregnancy is safe; however, the best methods for optimising this practice to improve blood pressure control and enhance maternal and foetal outcomes remain unclear. 
- The My Pregnancy Care intervention was co-designed and optimised to support women in self-managing their blood pressure during pregnancy in collaboration with healthcare professionals. Information, prompts and clear messaging were key features to support behaviour change. 
- The My Pregnancy Care intervention is designed to support behaviour change in women and healthcare professionals and has the potential to improve pregnancy outcomes.

## Introduction

Hypertension is common in pregnancy, affecting about one in ten women [1, 2]. Around half of pregnant women with hypertension will develop pre-eclampsia. This serious multi-system disorder is a major cause of maternal and perinatal death, foetal growth restriction, premature birth and stillbirth [3, 4]. Pre-eclampsia is characterised by raised blood pressure (BP) developing after 20 weeks gestation, accompanied by protein in the urine (proteinuria). Early diagnosis can reduce complications for both the woman and the baby, making regular BP and urine monitoring an important part of antenatal care [5].

BP self-monitoring allows frequent measurements to be taken with little disturbance to daily routines. It is commonly used in the management of hypertension among the non-pregnant population within primary and secondary care, proving safe, cost-effective, and significantly increasing the time BP remains within the target range [6, 7].

Currently, substantial resources are used to monitor pregnant women with hypertension, both from an individual and a National Health Service (NHS) perspective. Additional antenatal appointments are recommended to check BP, ranging from once or twice a week to every 4 weeks, depending on BP control and individual patient need [5]. This has led to the exploration of BP self-monitoring in this population [8, 9] to reduce the burden of multiple clinic visits for women and their healthcare teams [10], provide more accurate and representative data for clinicians to base treatment decisions on, and increase women’s involvement and engagement with their care.

Alongside BP measurement, proteinuria testing is also routinely undertaken to screen for the development of pre-eclampsia. During pregnancy complicated by hypertension, it is recommended by The National Institute for Health and Care Excellence (NICE) that this is performed once or twice a week, alongside BP checks. Using dipsticks to test urine for protein is simple, inexpensive and rapid, and research has shown that women can perform these tests as accurately as healthcare professionals [11]. Consequently, urine testing could form part of a self-monitoring regime in pregnancy, together with BP self-monitoring, to address the diagnostic aspects of pre-eclampsia [12, 13].

Whilst trials have shown that BP self-monitoring in pregnancy is safe, using it in clinical practice to improve BP control or pregnancy outcomes remains a challenge [9, 14, 15]. The BUMP 1 and BUMP 2 randomised control trials investigated BP self-monitoring in pregnancy, with participants inputting their readings to a smartphone application (app), which clinicians could review remotely or directly at a clinic appointment. They found that BP self-monitoring with tele-monitoring did not lead to significantly improved BP control or earlier detection of hypertension in pregnancy. Most women did use the intervention as intended and submitted daily readings, showing that BP monitoring alone is feasible. Previous work outside of pregnancy has shown that co-interventions, such as individually tailored support or personalised education alongside BP self-monitoring leads to larger reductions in BP than self-monitoring alone [16]. One way this can be implemented is by remote antihypertensive medication titration based on self-monitored readings. This has been shown to be effective at improving BP control in non-pregnant populations and in the postpartum period but is yet to be trialled in pregnant women [17–19].

### Aims and objectives

This study aimed to co-develop an intervention to facilitate effective BP monitoring, healthcare professional decision-making, and early detection of pre-eclampsia in hypertensive pregnancies. More specifically, the objectives were to refine a prototype intervention based on stakeholder input, ensuring that participants from diverse backgrounds were included to maximise the accessibility and efficacy of the app across the maternity population without amplifying existing inequalities.

## Methods

### The intervention

The prototype intervention included a multi-component app incorporating BP self-monitoring, proteinuria self-testing and remote antihypertensive medication titration, alongside written patient information and clinical responses to data submitted. It was based on an app and leaflet developed and tested by the BUMP trials on BP monitoring in pregnancy and previous qualitative and quantitative data on self-monitoring in pregnancy.

### Theoretical framework

This study follows the Medical Research Council (MRC) framework for the development of complex interventions, which treats intervention development as an iterative process based on empirical evidence and theoretical reasoning [20]. It prioritises stakeholder input, specifically from those with lived experience of hypertensive disorders of pregnancy, to ensure real-world applicability of the intervention [21, 22]. Empirical evidence includes existing literature and the researchers' prior work. Theoretical reasoning is guided by the COM-B model of behaviour change (capability, opportunity, motivation), which identifies barriers and facilitators to change. These can be addressed through key intervention features visualised in logic models. Where intervention features were directly related to a behaviour, behaviour change techniques derived from the behaviour change taxonomy were detailed in the logic model [23]. This is important in the reporting of behavioural interventions to improve clarity around the active ingredients leading to behaviour change that can then be replicated in future work [24].

### Patients, public contributors and stakeholders

Stakeholders included obstetricians, midwives, patient representatives, and researchers. The group, which has experience in pregnancy hypertension, digital health design, women’s health and hypertension, was invited to discuss current research findings and the wants and needs of pregnant women with hypertension and their healthcare professionals. Their input was invaluable in shaping the design of the initial materials.

Three key aspects were voiced in stakeholder discussions:

- Regular monitoring (taking regular measurements of BP or urine and promptly inputting results)
- Acting on readings appropriately (e.g. repeating/ seeking advice on abnormal readings)
- Medication self-management (aiding adherence and enhancing trust and communication between patients and healthcare professionals)

It was also felt that women's belief in the benefits of self-monitoring for their own and their unborn baby's health was crucial for their motivation to engage [12, 25].

The intervention from previous BUMP trials was used as a basic framework, with the look and navigation kept relatively unchanged as this was well accepted by previous participants. The intervention was designed pragmatically for women with either chronic or gestational hypertension (as the previous BUMP2 trial) after discussion with stakeholder groups. The three key aspects derived from stakeholder group discussions were incorporated, which formed the draft multi-component app intervention, named *My Pregnancy Care,* and the production of an intervention information leaflet. These were shared with study participants [22]. Patient representatives continued to support the research throughout.

### Ethical approval

Ethical approval for this study was given by the Research Ethics Committee North West - Preston Research Ethics Committee (22/NW/0175). The sites involved were Oxford University Hospitals NHS Foundation Trust and Manchester University NHS Foundation Trust.

### Recruitment

Women and healthcare professionals were recruited through two sites to provide views from different settings, and approval was obtained to recruit up to 20 in each group. Recruitment with purposeful sampling continued until a broad and diverse sample was obtained and data saturation was reached.

Participants were provided with information about the study and time to consider participating. Informed consent was obtained in person or verbally if interviews took place on the phone or online.

Purposive sampling was used to recruit 11 women with lived experience of hypertension in pregnancy at 2 NHS Trusts and via stakeholder charity and support groups. Women from diverse backgrounds and ethnicities were sought to ensure the voices of under-represented groups were included to maximise the feasibility, accessibility and efficacy of the multi-component app across the maternity population.

Eighteen healthcare professionals involved in caring for women with hypertension in pregnancy were recruited from two NHS Trusts. A range of experience in the use of BP self-monitoring and urine self-testing in pregnancy was sought within this sample. Verbal consent was gained from participants, with consent forms completed by the researcher.

### Data collection

Think-aloud interviews, a common qualitative interview technique for developing digital interventions, were conducted with participants who had experienced hypertension in pregnancy. This involved participants engaging with the proposed technology and verbalising their thoughts [26]. Participants were asked to consider the information materials, mock-ups of the app, scripts for reminder messages and recommendations in response to submitted BP and urine readings, including those recommending medication changes. They were also asked to consider the usability of the proposed tele-monitoring aspects. Open questions assessed how understandable, acceptable, relevant, and likely to elicit an appropriate response the materials were; whether they reassured, rather than raised concerns; and whether there were any obvious gaps in the materials planned. Women were asked to consider how they might act in hypothetical situations in response to readings or messages to explore a range of scenarios and situations.

Individual interviews and focus groups consisting of two to five participants were conducted with obstetricians and midwives involved in antenatal care, face-to-face or online (depending on the participant’s preference). These were semi-structured to allow a broad consideration of the intervention. The healthcare professionals were shown materials and messages and asked to consider hypothetical clinical situations, communication pathways and practicalities that would support appropriate clinical action, and to consider potential behaviour change facilitators that could be incorporated into the app [13]. Focus groups were used with healthcare professionals to allow interdisciplinary discussion, potentially leading to exploration of new areas. Recordings and notes from discussions were kept for analysis. Formal transcription was not undertaken (due to time and funding).

### Analysis

**Fig. 1:**
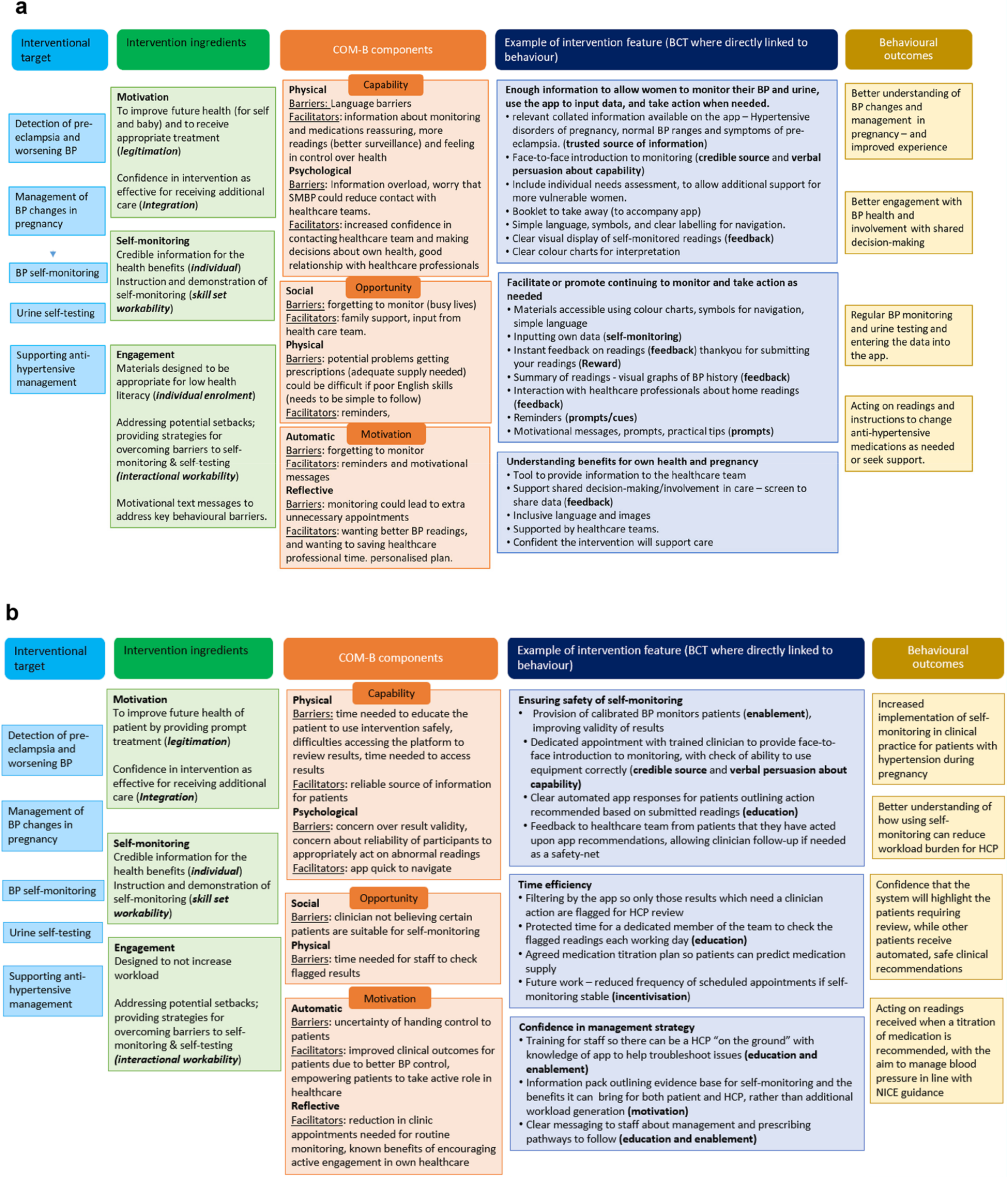
**a** Logic model using the COM-B model of behaviour change to identify how the intervention could support behaviour change in women with hypertensive pregnancy. **b** Logic model using the COM-B model of behaviour change to identify how the intervention could support behaviour change in healthcare professionals managing those with hypertensive pregnancy

A “table of changes” was created for both participant groups, organising positive and negative views to inform app development and added to iteratively. The views were coded as important for behaviour change, easy and uncontroversial, said repeatedly, supported by experience, does not contradict, not changed and not possible. A requested feature or aspect of the intervention could be given more than one code. The rationale for a change, either implemented or not, was documented and prioritised using the MoSCOW framework (must have, should have, could have, would have). The data were then organised into logic models using the COM-B model of behaviour change to identify how the app could support behaviour change (Fig. 1a and b).

## Results

The healthcare professional sample consisted of 18 midwives and obstetricians working in maternal health and medical research. Participants were at various stages in their career paths, with professional work experience ranging from 3 to 18 years (see Table 1). Specialist clinics were the most frequently reported usual work environment with antenatal clinic, postpartum clinical care and research also featuring highly. Some reported experience of using self-monitoring readings to guide management decisions for pregnant women, often in the context of white coat hypertension, where BP levels are elevated in clinic settings due to environmental factors.

**Table 1:** Participating healthcare professionals

Job title	Grade	Years of experience	Usual work environment
Obstetrician	ST2	3	Antenatal clinic, specialist clinic, postnatal ward
Obstetrician	Consultant	12	Antenatal clinic, specialist clinic, postnatal ward
Obstetrician	ST6	6	Antenatal clinic, specialist clinic, postnatal ward
Obstetrician	ST4	4	Antenatal clinic, specialist clinic, postnatal ward
Obstetrician	Consultant	15	Antenatal clinic, specialist clinic, postnatal ward
Midwifery lecturer/manager	Band 8	6	Research
Midwife	Band 6	10	Research
Consultant midwife trainee	Band 8a	9	Specialist clinic
Midwife	Band 6	9	Antenatal clinic
Midwife	Band 7	3	Antenatal clinic, research
Midwife/research midwife	Band 6	13	Specialist clinic
Midwife/research midwife	Band 6	8	Specialist clinic
Midwife/research midwife	Band 6	9	Specialist clinic
Midwife/research midwife	Band 6	7	Antenatal clinic
Specialist midwife—still birth	Band 7	4	Specialist clinic
Clinical research manager	Band 8	6	Research
Obstetrician	Consultant	10	Antenatal clinic, specialist clinic, postnatal ward
Obstetric physician	Consultant	18	Antenatal clinic, specialist clinic, postnatal ward

*ST* specialty trainee in a hospital specialty, where the number signifies the amount of years spent in training in that specific speciality

Eleven women with lived experience of hypertension in pregnancy, either currently or in previous pregnancies, also consented to take part. The sample included women from a diverse range of ethnic and socio-economic backgrounds including under-represented groups (see Table 2). All participants were familiar with BP self-monitoring, and most felt confident about the BP thresholds that would prompt them to seek advice from a healthcare professional. Most had bought their own BP monitor, some under the guidance of their antenatal clinical team but others without seeking any advice, whilst a few reported using a BP monitor already owned by a family member. Some of the women had used a simple logging app to record home readings that were then reviewed during clinic visits. None of the participants had prior experience of performing urine testing, though one of the patients and public involvement (PPI) representatives had.

**Table 2:** Participants with experience of hypertensive pregnancy

Participant	Age	Currently pregnant	First pregnancy	Experience of taking antihypertensive meds in pregnancy	Ethnic origin	Education level
1	25–34	Yes	No	No	Asian	Degree level or higher
2	35–49	No	No	Yes	Asian British Pakistani	School (A levels or equivalent)
3	35–49	No	Yes	No	White Irish	Degree level or higher
4	35–49	No	No	Yes	Gypsy or Irish Traveller	School (A levels or equivalent)
5	35–49	No	No	Yes	Gypsy or Irish Traveller	School (A levels or equivalent)
6	35–49	Yes	No	Yes	Black African	Degree level or higher
7	35–49	Yes	No	Yes	White British	Degree level or higher
8	35–49	Yes	No	Yes (only in a hospital setting)	Black Caribbean	School (pre-GCSE)
9	35–49	Yes	No	Yes	White British	Degree level or higher
10	35–49	Yes	Yes	Yes	Black British	Degree level or higher
11	> 50	Yes	No	Yes	Black African	Degree level or higher

**Fig. 2:**
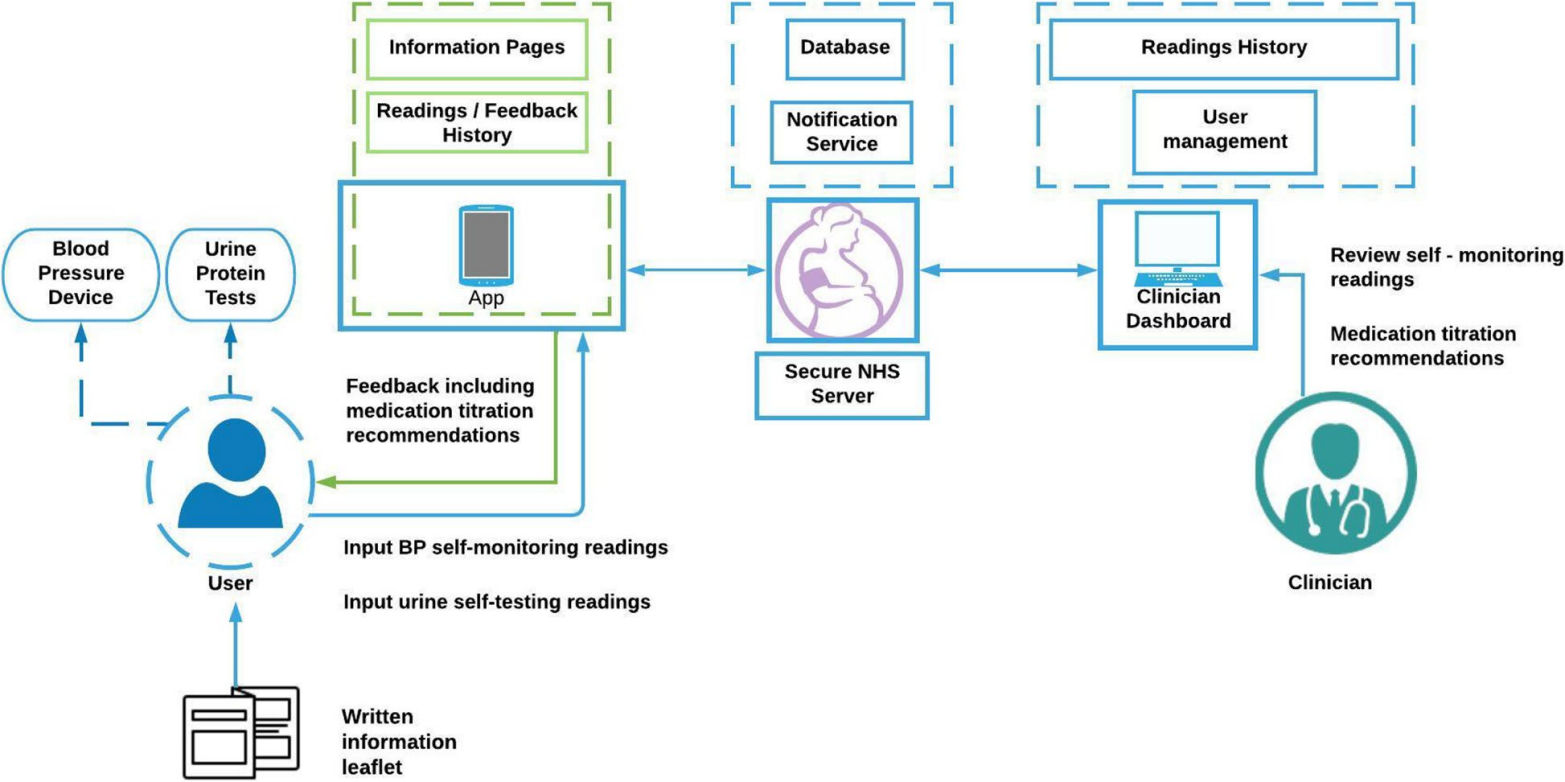
Intervention flow model for the My Pregnancy Care intervention

An overview of the final intervention is shown in Fig. 2. The details of the individual intervention features are outlined below.

### Regular monitoring of blood pressure and proteinuria

Overall, both groups were in favour of the concept of BP self-monitoring and urine self-testing during pregnancy. Daily BP self-monitoring was felt to be appropriate and achievable by women and healthcare professionals.

*“It would be useful to be part of the daily routine”* (participant 1).

Weekly proteinuria testing was also well received in principle by both participant groups.

*“I’d be happy to test urine if my BP was up, that would be ok, occasionally not every day”* (participant 2)

Additional urine testing alongside BP monitoring was also deemed acceptable. It was understood that this would be required to guide a suitable clinical response and, therefore, maintain patient safety.

Most women said they would prefer a daily check of their BP submissions by a clinician but accepted that readings from late evening or weekends may not be seen until the next working day. Women wanted guidance on when to seek urgent clinical review in these circumstances, with some feeling reassured by an on-screen cue indicating their readings had been reviewed. These views were mostly shared by women who had previously experienced poor pregnancy outcomes, reflecting the strong emotional drivers that may inform behaviour.

Healthcare professionals however felt it was unfeasible to have a clinician review all submitted self-monitoring results daily, especially if the intervention was scaled up across a maternity department. The design phase required balancing workload concerns with support strategies, as healthcare professional involvement was crucial to the intervention’s success. The role of prescribing midwives in triaging and managing results was discussed, but their limited availability made this an impractical universal solution.

### Relationship with healthcare professionals

Women were reluctant to reduce the number of standard clinic visits, as they valued relationships with their healthcare team, and did not want to feel that the app was used as a replacement, or would lead to less overall contact with healthcare professionals. However, most were willing to reduce extra antenatal visits required for hypertensive pregnancies.

“…as (it’s) just what happens with the clinic but would save the trips in.” (participant 7)

This view was shared by some healthcare professionals who did not want to “miss out opportunities to pick up other problems” and felt it was important that BP self-monitoring was used to “enhance not replace care”.

### Workforce burden

Concerns were raised by healthcare professionals about the time commitment and practicalities of checking submitted results from patients’ self-monitoring and were keen to ensure the intervention did not add to the workforce burden. They felt strongly that the responsibility should be with the app users, not clinicians, and that app users should seek medical review for abnormal readings to ensure timely action without adding to their workload. The reasons for this were twofold: ensuring patient safety with abnormal results being acted on in a timely manner and minimising potential to increase workload.

### Accessing readings in clinic

Access to self-monitoring readings during clinic appointments was important. Women wanted to easily show their readings to the clinician and clarify their medications, whilst healthcare professionals wanted quick access to recent results, BP trends and current medications. A suggested solution was a summary screen in the app displaying these features, including a graph to easily visualise BP trends, which could be accessed without a mobile signal.

“Staff need to have, would benefit from, seeing a woman’s history and context on the app” (Midwife 1)

### Intervention features designed to support regular monitoring

#### Automated responses and reminders

Thresholds for action were based on consultation with multiple clinicians. Each BP or urine result submitted received an automated response stating whether it was low, normal, raised or high and next steps the patient should take. This included suitable timeframes to seek review for abnormal readings, placing responsibility on the patient to act. The language of these messages was checked by participants to ensure they were clear and understandable. The app included expected timeframes for clinician review of results (usually those requiring consideration of a medication change). Only results requiring clinician action were highlighted to healthcare professionals and checked once daily during the work week.

Reminders for participants were also integrated into the app as these were considered important for habit formation.

“Reminders would be a really good idea…making it part of a routine” (Participant 4)

Reminder messages which were sent if no readings were submitted for three consecutive days were considered “a good prompt to carry on taking readings”, thereby improving adherence to the intervention.

#### Information for healthcare professionals

A concise information pack was produced for healthcare professionals, outlining how BP self-monitoring was safe and effective outside of pregnancy and highlighting its benefits, empowering women to become more involved in their care and collaborate in shared decision-making, enhancing understanding of BP and medications and reducing the burden of BP monitoring in clinic. It was anticipated that this would motivate healthcare professionals to engage with the intervention which in turn should facilitate use by patients, who expressed they would be happier if they felt the app was endorsed by their healthcare team. It was suggested that a member of the healthcare team act as an “app champion” at each site to provide ground support for users and help integrate the new tool into existing antenatal care pathways.

#### Information for women around blood pressure in pregnancy

Including clear information about BP in pregnancy in the app was considered important, with increased knowledge impacting on adherence to self-monitoring. Women primarily wanted information on antihypertensive medications in pregnancy (with an emphasis on safety for baby), pre-eclampsia and potential symptoms, lifestyle advice for managing BP in pregnancy and postnatal care (especially potential antihypertensive medications). There was a request for simple language to ensure accessibility and the option to provide information in different languages.

Healthcare professionals were keen to include information on the safety of BP medication in pregnancy, as they felt this would improve confidence in treatment options and, therefore, adherence. Some women and healthcare professionals suggested that an in-person introduction to monitoring and the equipment would be useful, supplemented by written guidance in a leaflet or the app.

Participants were asked to provide feedback on the clarity of included information. The women differed in regard to the amount of detail they wanted, so it was decided only the most relevant information would be conveyed in the app, with links to trusted external resources for women wanting additional information.

#### Motivational messages

Motivational messages were thought by most women to have a supportive role within the intervention, preferably a short weekly message. Consequently, weekly app messages about BP control and pregnancy related facts were developed. Example messages included “A great way to get into the habit of taking your BP is to choose a time to suit you and setting an alarm on your phone as a reminder” and “BP can change from one day to the next. By checking it at home you help your midwife take care of you”. These messages aimed to drive engagement with the intervention and promote positive behaviour change.

#### Acting on readings

In their prior use of BP self-monitoring, some participants believed their readings had been looked at by healthcare professionals every day or even as they were entered. However, focus groups and interviews with clinicians suggested that self-monitoring readings were usually only viewed retrospectively during appointments. This discrepancy highlighted the need for clear message responses stating that results would not be reviewed in real time, accompanied by information on who to contact, how to contact them and when to do so in response to an abnormal reading. To support decision-making, clinicians requested an interface offering easy access to patients’ results and current medication information in one place. It was important that this could be accessed without logging into a separate system and ideally linked to electronic health records.

#### Intervention features to support acting on readings

There was overlap between the intervention features designed to support regular monitoring and those to support acting on readings, particularly the automated instructions in response to submitted readings. The digital platform for the clinical team was designed to present all required information to guide management decisions on a single, easy-to-read display. The app could automatically update the patient’s medication information after a recommended increase by a clinician or by the patient themselves with a confirmation prompt—ensuring the information was up-to-date and accurate.

#### Designated healthcare professionals to check readings daily

Currently, clinicians must log into a separate system to view submitted readings. However, assigning healthcare team members to check flagged results at a set time each day could increase system familiarity and reduce frustrations around access. It would also ensure that protected time was dedicated to this aspect of patient care, rather than it becoming absorbed into the general workload, reducing barriers to engagement.

#### Confirming that messages had been received by women

An additional safety net was incorporated by the inclusion of a check box on app messages requiring women to acknowledge receipt of information and that they would follow any advice given. If these were not completed within a certain time period it would generate a flag, prompting clinician review. This aimed to ease clinician concerns about women responding appropriately to abnormal readings (a potential barrier to behaviour changes and intervention use).

#### Self-management of antihypertensive medication

Few women felt completely comfortable taking antihypertensive medication during pregnancy, but all agreed it was important for their health, and their baby’s health if it improved BP control. All were happy to complete remote medication changes based on their home readings when a “clinician check” took place (a medication increase approved by a clinician after reviewing results remotely). Many were also happy for a titration step between in-person clinic reviews, which was pre-agreed.

“I'm happy to use the app, but I would want someone to check[before medication titration]” (Participant 3)

Similarly, healthcare teams were supportive of at least a single step medication titration being performed remotely, with most happy for this to be pre-agreed and without further clinical checks (given that very high readings would prompt a clinical review first). There were concerns raised about “app fatigue” from healthcare teams, as several apps including BP and gestational diabetes monitoring apps are already available and sometimes used in practice, which could be a barrier to healthcare professional engagement.

#### Intervention features to support self-management

Women's desire for a “clinician check” before making medication changes was a recurrent theme, alongside the need for clear guidance relating to medication changes for both women and healthcare professionals. Limiting remote changes to a single incremental change in a medication already prescribed removes barriers to implementation, including difficulties in obtaining new prescriptions in a timely manner. Having a pre-agreed medication titration plan between patient and clinician was felt to be likely to improve adherence and acceptability.

For healthcare professionals, messages relating to recommended medication titration were framed around the NICE guidance and its targets for BP in pregnancy. This was to increase confidence, reassuring clinicians their actions are evidence-based.

### Areas of difference between the user groups

#### Reporting symptoms

Several women felt their concerns had gone unheard when reporting hypertension-related symptoms and suggested the intervention could be used to ensure clinician acknowledgement in future. Whilst happy for the intervention to provide information about symptoms in pregnancy, healthcare professionals were wary of collecting symptoms inputted by women. They expressed concern that women may feel reporting via the app was all the action they needed to take, which could lead to delayed detection of complications. As a compromise, clear information about symptoms that would be important to act on was included, alongside explanations for women about how to escalate concerns.

#### Peer support

Several women said that support from others in a similar situation would be valued, potentially via a forum on the app. Whilst this was not included in the final intervention, it may be possible to provide links to outside groups where peer support could be accessed in future.

## Discussion

### Main findings

This multi-component intervention was developed by building on previous work and actively engaging key stakeholders in its iterative design to ensure that both women and healthcare professionals have the capability, opportunity and motivation required for effective self-management of blood pressure during pregnancy.

Participants agreed that the proposed app and supporting materials were feasible in principle. Suggested changes made the intervention more acceptable, understandable and safe for future implementation. The fact that most participants were already self-monitoring, often on their own initiative, further demonstrated their motivation. A key component involved supporting women to respond appropriately to abnormal readings. Automated in-app responses with simple language to guide next steps, proven effective in previous digital interventions like post-natal hypertension self-management, were considered integral.

The intervention’s ability to facilitate prompt medication changes without increasing healthcare professional’s workload was a crucial factor in its acceptability. Educational features and clear guidance pathways provide an opportunity to reduce some clinic visits whilst maintaining the important clinician–woman relationship. The importance of this relationship is echoed in other reports and highlights the role of social opportunity in facilitating behaviour change, where healthcare professional support is essential in empowering women to self-manage their health during pregnancy [27].

### Clinical context

Other research has shown that self-monitoring of blood pressure in pregnancy is already widespread [28–30]; however, the readings tend to be reviewed by clinicians only on the day of remote appointments or during clinic visits. Consequently, the data are not used pro-actively, and opportunities to optimise BP control and enhance interactions between healthcare teams and women are being missed.

A simple system to facilitate medication changes could support healthcare professionals in up- or down-titrating BP medications when needed. Ensuring the intervention integrates smoothly into existing pathways and is user-friendly for both women and healthcare professionals is crucial. Core features of this intervention include messages and information that guide and empower users to act appropriately and promote collaborative, shared decision-making.

From the COM-B model perspective, we can see that “Capability” is enhanced through education, user-friendly design, and clear guidance. This provides an “Opportunity” to enhance current self-monitoring practices, reduce clinic visits whilst maintaining the clinician–woman relationship and integrate the proposed intervention smoothly into existing healthcare pathways. “Motivation” is strengthened by empowering women with knowledge, providing automated feedback, and maintaining healthcare professional support. The next step is to trial the multi-component app on a small scale to assess its feasibility in clinical practice.

### Strengths and limitations

The study benefited from the strong starting point of a well-designed and trialled intervention of BP self-monitoring alone and a highly experienced expert group, up to date with the latest research findings and theory. This supported the initial development of draft materials that were used in the iterative development. The iterative development of the app focused on involving women with lived experience of hypertension in pregnancy and the types of healthcare professional they interact with most, with a further strength being the representation of under-served groups. Use of behaviour change theory strengthens the data generated within the study by identifying and then prioritising the views and perspectives of these groups, improving the capabilities, opportunities and motivation of key stakeholders to engage successfully with the developed intervention.

Limitations include the recruitment from two main hospital sites where self-monitoring of blood pressure is already used in some capacity, which may have affected the data and made it less representative for regions where this is not currently used. The wider expert team (including PPI) involved individuals from several other regions and settings to help mitigate this bias. All women recruited, except for one, were over the age of 35, which could reduce the generalisability of findings to a younger pregnant population**.**

## Conclusion

BP self-monitoring is an accepted part of antenatal care; however, its current use does not support prompt actions based on readings taken between clinic appointments. Timely incorporation of self-monitored data via a multicomponent app could improve BP control and pregnancy outcomes. Both women and healthcare professionals viewed self-monitoring and self-testing positively, appreciating its potential to improve relationships, women’s understanding of their BP, and overall care and pregnancy outcomes. Remote titration of antihypertensive medication based on BP self-monitoring readings was generally acceptable, with the caveat that it includes a clinical check, and that titration was limited to already prescribed drugs. Consolidating self-monitoring data and trusted information in one place was considered beneficial. The next steps include a small-scale feasibility trial to assess the designed intervention’s feasibility in clinical practice.

## Data Availability

Data from this study will be made available upon reasonable request, subject to the approval of the sponsor (University of Oxford).
